# Species-Specific Diversity of a Fixed Motor Pattern: The Electric Organ Discharge of *Gymnotus*

**DOI:** 10.1371/journal.pone.0002038

**Published:** 2008-05-07

**Authors:** Alejo Rodríguez-Cattaneo, Ana Carolina Pereira, Pedro A. Aguilera, William G. R. Crampton, Angel A. Caputi

**Affiliations:** 1 Department of Integrative and Computational Neurosciences, Instituto de Investigaciones Biológicas Clemente Estable, Montevideo, Uruguay; 2 Department of Biology, University of Central Florida, Orlando, Florida, United States of America; Smithsonian Institution, United States of America

## Abstract

Understanding fixed motor pattern diversity across related species provides a window for exploring the evolution of their underlying neural mechanisms. The electric organ discharges of weakly electric fishes offer several advantages as paradigmatic models for investigating how a neural decision is transformed into a spatiotemporal pattern of action. Here, we compared the far fields, the near fields and the electromotive force patterns generated by three species of the pulse generating New World gymnotiform genus *Gymnotus*. We found a common pattern in electromotive force, with the far field and near field diversity determined by variations in amplitude, duration, and the degree of synchronization of the different components of the electric organ discharges. While the rostral regions of the three species generate similar profiles of electromotive force and local fields, most of the species-specific differences are generated in the main body and tail regions of the fish. This causes that the waveform of the field is highly site dependant in all the studied species. These findings support a hypothesis of the relative separation of the electrolocation and communication carriers. The presence of early head negative waves in the rostral region, a species-dependent early positive wave at the caudal region, and the different relationship between the late negative peak and the main positive peak suggest three points of lability in the evolution of the electrogenic system: a) the variously timed neuronal inputs to different groups of electrocytes; b) the appearance of both rostrally and caudally innervated electrocytes, and c) changes in the responsiveness of the electrocyte membrane.

## Introduction

Fixed motor patterns are discrete motor patterns that, when switched on, produce well defined and, coordinated movements or activities. The term “fixed” implies that the patterns of activity are stereotyped and relatively constant within and among individuals of a given species [1]. The electric organ discharges of weakly electric fish are species-specific fixed motor patterns coordinated by spinal and peripheral mechanisms [2], [3], [4]. Electrogenesis offers several advantages as a paradigmatic model of fixed motor pattern. Thus, for example, the four questions that Tinbergen [5] thought should be asked of any behavior can be readily investigated with respect to the electric organ discharge, as described below. Tinbergen's four questions concerned: a) organization and causation, b) development, c) function, and, d) evolution of the neural and peripheral structures subservient the fixed motor pattern.

a) Organization and causation: The electromotor system is well suited for analyzing neural coordination mechanisms. Its simplicity allows one to integrate knowledge obtained using different approaches, and to investigate the system at different levels of organization. Work over many years has shown that the electric organ discharge of pulse gymnotiforms results from the transformation of a single neural impulse originating from a synchronous pacemaker into a pattern of electromotive force which results from the sum of action currents generated by electrogenic cells (electrocytes) [2], [4], [6]. This knowledge led to the development of 1) non-invasive techniques permitting the characterization of the electromotor system in intact live fishes [7], [8], and 2) computational models for calculating the electric field from these measured parameters [9], [10].

b) Development: Descriptions of the electric field in the larvae and juveniles of various species are available from studies in the wild [11] and in captivity [12]–[18]. Moreover, complete descriptions of the electric organ, the electromotive force, and electric field generation in the model species *Gymnotus omari* sp. nov. from Uruguay (formerly identified as *G. carapo*) allow one to explore the developmental mechanisms responsible for the diversity exhibited by adult phenotypes [18].

c) Function: The spatiotemporal pattern of electromotive force generated with each electric organ discharge is the ultimate cause of the electric fields that serve as carriers for electrolocation [19], [20] and electrocommunication signals [21]. Extensive reviews of electrolocation and electrocommunication have been edited by Fessard [22]; Bullock and Heiligenberg [23] Moller [24] and Bullock, et al. [25] It has been speculated that the species specificity of the electric organ discharge is important in enhancing the signal to noise ratio of self generated signals [26]. This speculation is based on i) a stereotyped waveform of the carrier which determines and restricts the range of capacitances that modifies the local electric organ discharge waveform [27]–[30]; and ii) the observation that the frequency band of electroreceptor response matches the power spectral density histogram of the electric organ discharge associated field [31]–[33]

d) Evolution: Local communities of weakly electric fish usually exhibit several species [34]–[38].The analysis of the local carrier, lead to the hypothesis that the electric organ discharge waveform generated by caudal region of pulse gymnotids constitutes itself a signal for recognition of conspecifics [39]. Therefore, electric organ discharges are suspected of playing an important role in reproductive isolation among incipient or fully-formed species, and hence the mechanisms underlying the origins and maintenance of species diversity [38], [40]–[42].

In *Gymnotus*, the most diverse and widespread genus of the New World order Gymnotiformes [42], the complexity of the electric organ discharge requires separate but coordinated activation of the different electrocytes (effector cells) along the length of the fish. This indicates that the nervous system generates a more complex pattern of output in controlling the electric organ discharge than is present in other electric fishes, including both Mormyridae (from Africa) and other gymnotiform fishes [4], [6].

Species characteristic variations in *Gymnotus* electric organ discharges suggest that changes in the effector electric organs or their motor control systems accompany species-level diversification. *Gymnotus* therefore represents a promising model taxon for exploring the electromotor system correlates of signal diversity. For these reasons a comparative study of *Gymnotus* is expected to yield unique insight into the expression of neural and peripheral devices that accompany species-level variation in electric organ discharges.

This paper is the first of a series aiming to identify commonalities and differences among the species-specific electric organ discharges in the genus *Gymnotus*. Here we focus on the characterization of the spatiotemporal fields and patterns of electromotive forces expressed by three species, *G. carapo, G. coropinae, and G. omari.* The first two are common tropical species, distributed over large areas of northern South America, whereas the last one, *G. omari* is a temperate species from Uruguay. Electrogeneration mechanisms have been most extensively studied and are best known in *G. omari* [4], [6], [43]–[51].

## Results

### The far-field head-to-tail potentials

**Figure 1:**
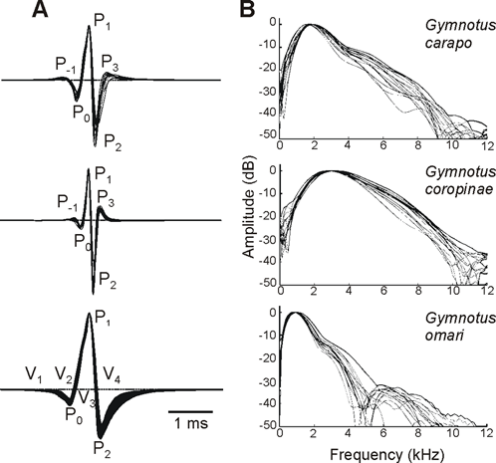
Head to tail electric organ discharges. Electric organ discharge waveform (a) and spectral power density (b) for 15 adult specimens of *Gymnotus carapo, G. coropinae* (both from Surinam), and *G. omari* (from Uruguay). Electric organ discharges were plotted with head positivity upwards, normalized and aligned to the peak amplitude of the dominant positive peak (P_1_). Scale bar = 1 ms. We indicate here the wave components using the two nomenclatures available from the literature. The nomenclature by Trujillo-Cenòz et al. [44] is based on the ordinal number of wave components (labeled as V) in the sequence of deflections observed at the head to tail recordings. These components were defined not only by their presence in the head to tail recordings but also by their different origin and mechanisms of generation [6]. Crampton's [38] nomenclature (used in several species of the genus) refers only to the ordinal number of each peak (P) in the head to tail recordings, referring to P_1_ as the main positive peak. For *G. omari*, P_0_ = V_1_ + V_2_, P_1_ = V_3_, P_2_ = V_4_. The application of a wave components based nomenclature to the head to tail recordings of *G. carapo* and *G. coropinae* is impossible because head to tail peaks are just the weighted sum of several waveform components of different origin, and probably generation mechanisms, which occur overlapped in time. Instead, we introduce a new nomenclature with a numeral sub index indicating the temporal order and a literal sub index indicating the spatial origin (r for rostral, c for central, and t for tail, see Fig. 5 for the pattern of electromotive forces and Fig. 8 summarizing our hypothesis on the electric organ discharge generators)

The far-field electric organ discharge waveforms of adults from the three studied species are illustrated in figure 1. *G. omari* generated a triphasic electric organ discharge beginning with a long head negative deflection (comprising two distinct components, that Trujillo-Cenòz et al. called V_1_ and V_2_ [44] and terminating with a second long head negative deflection (V_4_ [44]). Pulse duration varied from 2.603 to 3.687 ms (mean 3.17, n = 15), and the mode of the spectral density histogram (here referred to as peak power frequency) from 0.793 to 1.0185 kHz (mean 0.866, n = 15, 27°C).

*G. carapo's* and *G. coropinae's* electric organ discharges are substantially shorter lasting, comprising a series of deflections of successive opposite polarity. These deflections are small at the beginning, increase up to a maximum and decrease at the end. These deflections were called by Crampton [38] according to their ordinal position in the sequence referring always to the main positive peak P_1_. The electric organ discharges of *G. carapo* last 1.764 to 2.184 (mean 2.016 ms, n = 15, 27°C), while those of *G. coropinae* were approximately half this duration (range 0.928–1.455 ms, mean 1.121 ms, n = 15). This was reflected by species-specific ranges of peak power frequency (*G. carapo*: mean 1.856, range 1.606 to 1.961 kHz, n = 15, and *G.coropinae*: mean 3.014, range 2.759 to 3.246 kHz, n = 15, 27°C, Fig. 1).

To compare the amplitude of the signals, specimens of *G. carapo*, *G. coropinae* and *G. omari* were recorded at a standardized conductivity (30 µScm^−1^) and temperature (24°C) from the center of a 48×28 cm aquarium filled to 4 cm (with fish oriented parallel to the longest side of the tank). Energy of the far field recordings was evaluated by the root mean squared value of a 10 ms trace (Table 1). All specimens of *G. omari* generated stronger fields than those generated by *G. carapo* and these in turn were larger than those generated by *G. coropinae*. Because the specimens of *G. coropinae* were much smaller than those of *G. carapo* (see methods) we normalized the data using the quadratic rule relating length and field amplitude found in *G. omari* [18]. The relationships between rms values still hold after normalization (Table 1; Mann Whitney/Wilcoxon test for non paired samples, p<0.01).

**Table 1:** Top row: Comparison between rms values of the head to tail electric organ discharge as measured in the same tank (45×26×4) at the same water conductivity and temperature.

	*G. omari* (N = 10, length between 11.6 and 22.5 cm)		*G. carapo* (N = 6, length between 20.6 and 29.8 cm)		*G. coropinae* (N = 12, length between 9.9 and 15.3 cm)	
	Median	IQR	Median	IQR	Median	IQR
rms value (mV/cm)	40.28	37.56	22.92	4.7	2.59	1.4
normalized rms (mV/cm^3^)	0.15	0.08	0.051	0.013	0.018	0.005

Bottom row: the same individual values were normalized by the square of the fish's length according to the findings of Pereira et al [18]. Columns: median and interquartile range for each species. All these fish were recorded between 10 to 20 days after capture.

### Analysis of the near-field potentials

**Figure 2:**
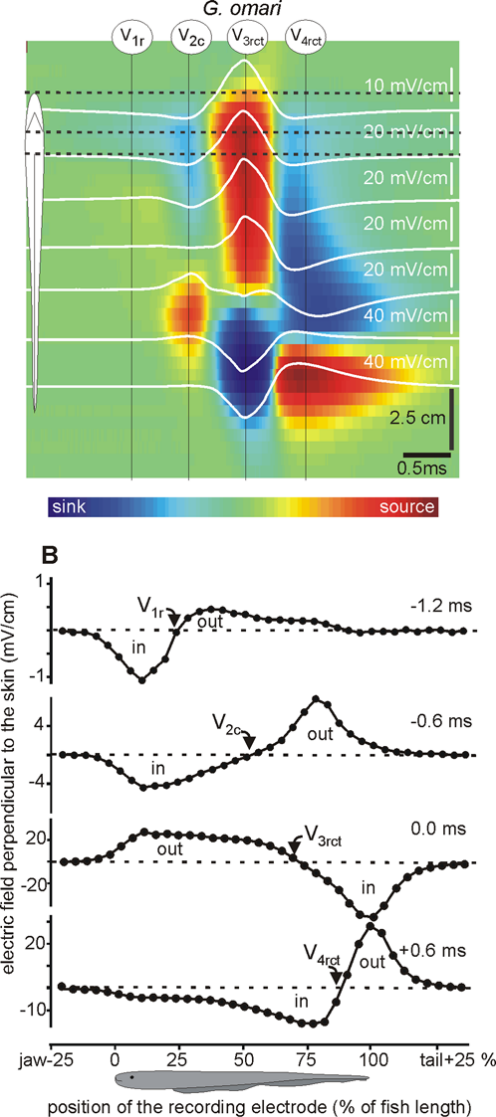
Transcutaneous current pattern of *G. omari*. Electric field perpendicular to the main axis of the fish body was recorded along a line 2 mm parallel to the mid-flank of *G. omari*. White traces correspond to the recorded field at equally separated points 2 mm from the skin on the side of the fish. These traces are superimposed on a color-map indicating the location of the sources (yellow-red-brown) and sinks (sky blue-deep blue) along the fish body (vertical axis) as the electric organ discharge progresses in time (horizontal axis); greenish correspond to negligible fields. *G. omari* shows 4 main components: V_1r_ inverting about the origin of the anal fin; V_2c_ inverting about half of the body; V_3rct_ and V_4ct_ inverting at the tail region. In this case there is a close correlation between temporal order and spatial origin of the components. For the sake of generality we used numeral sub index indicating the temporal order and a literal sub index indicating the spatial origin (r for rostral, c for central, and t for tail).

**Figure 3:**
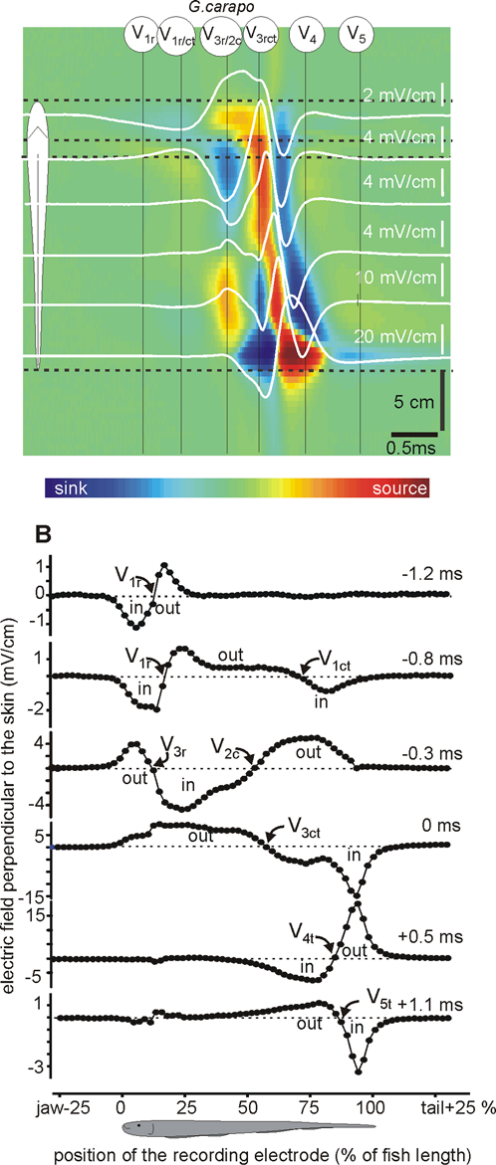
Transcutaneous current pattern of *G. carapo*. Electric field perpendicular to the main axis of the fish body was recorded along a line 2 mm parallel to the fish side of *G. carapo* (same resolution and color code as in Figure 2). This species shows the more complicated generator. We refer to each generator referring to its temporal order (numeral sub index) and spatial origin (literal sub index, r for rostral, c for central, and t for tail). Times are referred to the positive peak (0 ms). The first observed activity is caused by a much localized sink at the head and a source distributed on the abdominal region (reversal point about 10–15% of the fish length from the jaw, V_1r_). It is followed by a distributed activity corresponding to more than one generator. In fact, two sinks at the head and at the tail regions indicate two simultaneous spots of activity in the electric organ at about 0.8 ms before the positive peak (reversal points about 20 and 75%,). Half a ms later the rostral activity reverses direction while at the central region a more distributed sink (having its source at the tail) develops. The complex V_34ct_ (labels: 0 and 0.5 ms) has the same profile as in the other species and finally the electric organ discharge ends with a rebound (label: 1.1 ms, V_5_).

**Figure 4:**
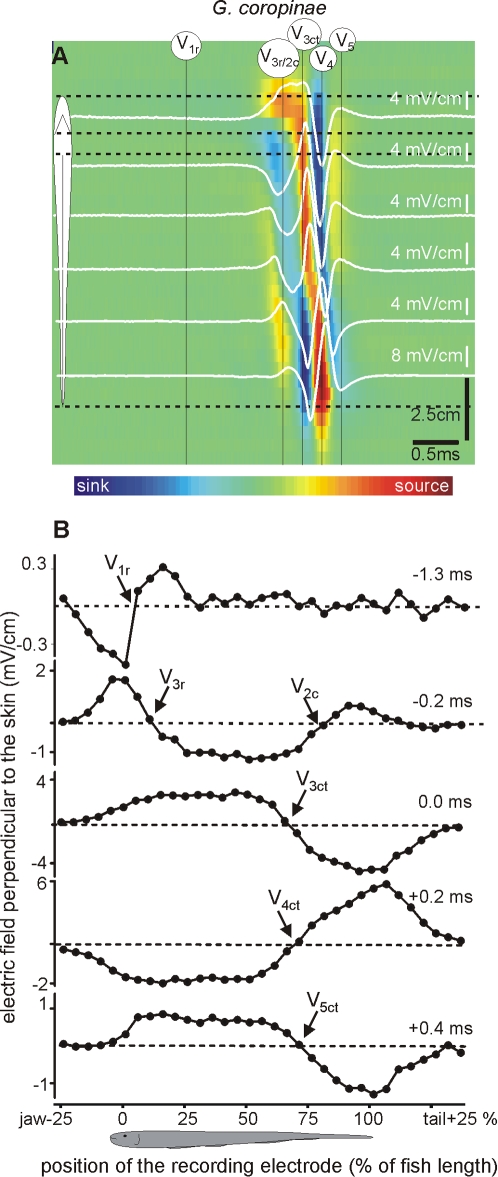
Transcutaneous current pattern of *G. coropinae*. Electric fields perpendicular to the main axis of the fish body was recorded along a line 2 mm parallel to the fish side in *G. coropinae* (same resolution and color code as in Figure 2). We refer to each generator referring to its temporal order (numeral sub index) and spatial origin (literal sub index, r for rostral, c for central, and t for tail). Times are referred to the positive peak (0 ms). This species is characterized by a first observed activity caused by a much localized sink at the head region (note that the reversal point, rostral sink-caudal source, 1.3 ms before the head to tail peak is located almost about the rostral pole of the fish). After a relative long period of time (−0.2 ms) this wave is followed by two spots activity indicated by the presence of two sources (at the head and at the tail) draining the current that sinks along the central body region. Shortly after the above, the fish's body becomes active. The complex V_34ct_ (0 to 0.2 ms) has the same profile as in the other species. Finally the electric organ discharge ends with a small rebound wave (0.4 ms, V_5_).

Gymnotiform fishes exhibit several generators that are better revealed by analyzing the near field potentials caused by the electric organ discharge [2], [6], [52]. Analysis of the perpendicular components of the electric organ discharge associated electric fields illustrated how currents flow inward and outward from the fish body and showed, unequivocally, the presence of multiple generators. Therefore, two local components of the near electric field (perpendicular and parallel to the main axis of the fish body) were simultaneously recorded along a parasagittal line passing 2 mm from the nearest point on the skin (Fig. 2, 3 and 4).

Each of the studied species exhibited characteristic strengths, activation timings, and locations of the electric organ discharge components. Several wave components of opposite polarity were generated concomitantly at different regions of the fish body. This finding obliged us to unify and make explicit the nomenclature of electric organ discharge components-taking into account not only the order of appearance of a component in the electric field recordings (V_1_–V_4_ as described by Trujillo- Cenòz [44] et al. or P_0_–P_2_ as described by Crampton and Albert [38], [42] ), but also its spatial origin. We maintained the traditional V labeling, and introduced a sub index letter to indicate the spatial origin of the component (r for rostral, c for central, and t for tail), including a sub index to indicate its order.

Selected recordings of the perpendicular fields were superimposed (white traces) on color-maps in which all the recorded traces were represented (Fig 2A, 3A and 4A). In these maps, the spatial (vertical) and temporal (horizontal) coordinates were represented in the axes of the map and the amplitude and direction was color coded (inward blue, outward red). The color maps' spatial dimensions and the baseline of the traces were located at the corresponding position, coded by the schematics of the fish body. Color scales were monotonically non-linearly adjusted in each case to maximize contrast. They provide a qualitative notion of the strength and timing of the sources (yellow-red) and sinks (sky blue-deep blue) complementary to the other plots. These color patterns suggested precise sequences of sinks and sources characteristic of each species. Rather than having a perfect synchronism, there was a general rostro-caudal shift in the reversal point as the activation progresses in time (Fig 2B, 3B and 4B).

Consistent with previous results [44], *G. omari* exhibited four main electric organ discharge components (Fig 2). i) An early slow head negative wave component generated at the abdominal region, referred to as V_1r_ with the sink-source reversal point (indicated by an arrow) near the anterior end of the anal fin (upper plot, Fig 2B). ii) A sharp head negative component, referred to as V_2c_, with a sink-source reversal point about halfway along the body and peaking 0.6 ms before the positive peak of the head to tail field (second plot, Fig 2B). iii) This component partially overlaps in time with the initiation of the sharp and head positive spike at the rostral regions. iv) This positive spike, which is the largest wave component, generated all along the fish's body. The duration of its wave subcomponents (rostral, V3r; central V_3c_; and tail V_3t_) are longer than the time shift between them and therefore they are substantially overlapped. Its reversal point at a time where all the electric organ is activated occurs at a distance from the anterior tip of the body, (p) equal to 70% of the fish body length (indicated by an arrow V_3rct_ third plot, Fig 2B). v) At decay phase of the positive component of the head to tail electric organ discharge the V_3t_ component overlaps in time with the starting of a late head negative component at the head region (V_4r_). vi) This late negative component propagates rostro caudally along the fish body. vii) Its larger generator is at the tail region, peaking about 0.6 after the positive peak of the head to tail field with a reversal point at p = 85% (V4_rct_, bottom plot, Fig 2B).

*G. carapo* exhibited a more elaborated generator (Fig. 3). i) An early slow head negative wave component was caused by a very localized sink at the head, and a source distributed over the abdominal region (reversal point about *p = *10–15% , V_1r_, Fig. 3B, maximum at 1.2 ms before the head to tail positive peak). ii) About 0.4 ms later, two separated sinks at the head and at the tail regions occurred, indicating the simultaneous activation of two widely separated generators in the electric organ (reversal points at *p* = 20 and *p* = 75% respectively, 0.8 ms before the head-tail positive peak). The caudal component partially coincided in time with V_1r_, but the field direction was opposed, with inward currents at the tail opposing outward currents at the central region. These early waves demonstrate the necessity of a nomenclature extension: V_1r_ (referring to the rostrally generated and head negative component present in all species) and V_1ct_ (referring to the early head positive component in *G. carapo*). iii) About 0.3 ms before the head to tail positive peak, a head generator caused a source at the rostral pole (V_3r_), while a central generator caused a simultaneous source at *p* = 75% of the fish length (V_2c_). Both generators overlapped (between *p* = 15 and *p* = 50%. iv) Finally, three components of the main complex (V_345_) are activated in a rostro-caudal sequence as if they were part of a positive-negative-positive wave that apparently propagates rapidly from head to tail (similar to V_34_ in *G. omari*).

*G. coropinae* had an electric organ discharge that was similar but shorter than that of *G. carapo.* The different components as analyzed by transcutaneous current flow included (Fig. 4): i) A very localized source-sink pair in the head region (V_1r_) that occurred 1.3 ms before the head to tail positive peak indicating the presence of a generator at the head region. ii) A very weak head-sided source-sink pair with a reversal point at P = 80% was present in some fish (V_1t_) 0.3 ms before the positive peak of the head to tail recordings (not shown). iii) Just after (0.2 ms before the head to tail positive peak) this wave was followed by two spots of activity indicating the presence of two sources. These two sources, one located at the head for V_3r_ and the other at the tail for V_2c_, drained the current that sank jointly at the central body region. iv) This was followed by a complex V_345ct_ that apparently propagates from head to tail in a similar way than V_34_ in *G. omari* and V_345_ in *G, carapo.*

### Analysis of the equivalent electromotive force generated by the fish's body

**Figure 5:**
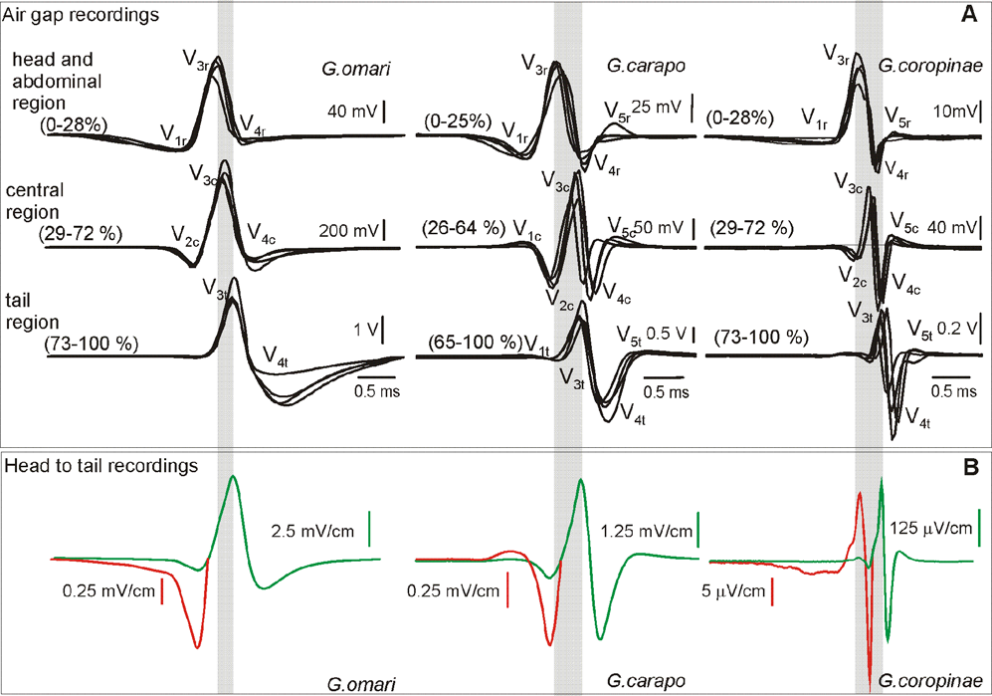
Electromotive force pattern of the three species. A) The air gap technique shows regional variations in the electric organ discharge electromotive force pattern. Note the presence of a smooth negative component and the advance of the main positive peak in the rostral regions of all fishes (marked by the widths of the gray bands), and the differences in the total duration of the electric organ discharges. B) A head to tail recording of the field between two electrodes separated 40 cm is shown below the air gap recordings (gray traces) to show the correspondence between the different deflections. Insets in red show a magnified version of the same waveforms for better showing the small early components. Note that different components of opposite polarity generated at different regions may overlap in time. For the sake of generality we name these components with a numeral sub index indicating the temporal order and a literal sub index indicating the spatial origin (r for rostral, c for central, and t for tail).

The external electric field results from an equivalent electromotive force acting on a distributed external load. Since this electric source is distributed, we estimated the equivalent electromotive force of three adjacent portions of the fish's body. To do so, we recorded the voltage drop between the boundaries of these portions when the fish was maintained in air (Fig. 5A). The rostral body portion comprised the head and abdominal region (the rostral 28%, in *G. coropinae* and *G. omari* and the rostral 25% in *G. carapo*) and the caudal body portion comprised the tail region (the caudal 28%, in *G. coropinae* and *G. omari* and the caudal 35% in *G. carapo*) while the central portion comprised the middle region of the fish body. In the three species the peak value for each component of the regional electromotive force profiles increased from head to tail (Table 2). The head to tail electric field was plotted below the air gap results in order to compare and relate the different electromotive force wave components with the field components that they generate (Fig 5 B).

**Table 2:** Peak amplitude of the electromotive force for every regional component of the electric organ discharge in the three compared species (mean±standard error).

	*G. omari* (mV)	*G. carapo* (mV)	*G. coropinae* (mV)
	Mean±std error, N = 12	Mean±std error, N = 4	Mean±std error, N = 8
V_1r_	−27.5±4.1	−15.1±5.3	−2.6±0.3
V_1c_	–	14.1±11.2	–
V_1t_	–	16.3±8	–
V_2_	−161.1±32.7	−86.1±29.8	−6.3±2.3
V_3r_	131.6±16.4	47.8±17.3	31.1±2.8
V_3c_	748.3±176.4	105.2±15.5	76.0±7.13
V_3t_	2346.3±394.9	1294.3±314.6	277.1±24.6
V_4r_	−12.8±4.0	−16.9±4.3	−11.3±0.8
V_4c_	−179.1±81.3	−78.2±23.4	−78.5±7.54
V_4t_	−1666.9±448.4	−1568.6±337.8	−387.7±42
V_5r_	–	2.5±5.0	0.4±0.1
V_5c_	–	10.7±2.8	6.8 ±1.6
V_5t_	–	158.8±51.2	30.2±5.5

The electromotive force profile corresponding to each portion of the fish body in *G.carapo* and *G. coropinae* can be described as variations on the basically similar pattern observed in *G omari*.

**Figure 6:**
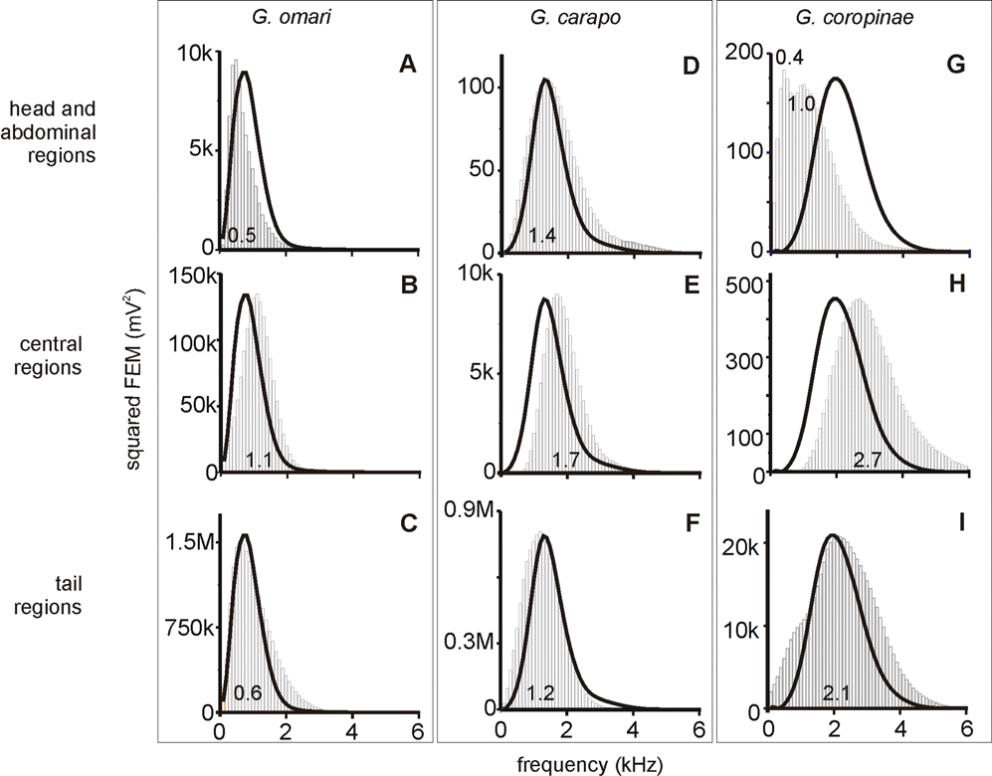
Different regions have a different spectral density. Power spectral density histograms showing that in all species the highest frequency wave components are generated at the central region of the fish's bodies. The modes of these histograms (peak power frequencies) are indicated on each plot. The black curves superimposed to the histograms are the envelopes of the power spectra of the head to tail electric organ discharge of the same fish normalized to the peak of the histogram for shape comparison. Note that in the two tropical species (*G. carapo* and *G. coropinae*) the power spectrum is shifted to a high frequency range, and that *G. coropinae* has two modes at the rostral region.

Among the commonalities we observed were: i) At the rostral regions, the electromotive force pattern consisted of a smooth slow head negative wave, followed by a fast positive-negative spike (V_13r_); ii) At the central region, a triphasic complex (V_234c_), with positive and late negative components of the complex V_34_ increased as a function of the generator distance from the tip of the lower jaw (Fig 5A); iii) Likewise, at the tail region, a biphasic complex (V_34t_) shows a large peak to peak amplitude (Fig 5A) iv) The duration of the main positive wave generated in the rostral regions (V_3r_) was longer than those generated in rest of the body (V_3ct_); this feature, associated with the smoothness of V_1r_, accounts for the lower peak power frequency of the electric organ discharge in the head and abdominal region (Fig. 6). v) The multiphasic pattern generated at the central region contributed very substantially to the whole head to tail electric organ discharge associated field, being largely responsible for the interspecific differences in peak power frequency (Fig. 6). vi) In all species was observed a time gap between the activation of the positive spike at the abdominal region and the activation of the positive spike at the central and tail regions of fishes' bodies (compare the timings of the peaks of V_3ct_ and V_3r_ ; Fig. 5 limits of the gray bands).

The main differences between the rostral electric organ discharges were: i) The amplitude of V_1r_ was largest in *G. omari*, intermediate in *G. carapo*, and smallest in *G. coropinae*; while its duration was significantly shorter in *G. carapo*. ii) In many specimens of *G. coropinae* there was a notch in V_3r_ indicating the presence of two distinct generators for this component in this region: one early, relatively smooth and long, and another sharp, similar in duration to V_3ct_. iii) While the late negative spike (V_4r_) was relatively large and sharp in the two tropical species, it was very small (if present) in *G. omari*. iv) A small V_5r_ was observed in some individuals of the two tropical species (Fig., 5A top traces).

There were three main differences between the waveforms originating from the central and tail regions of the fish body.

First, the duration of the components generated at the central and tail regions was largest in *G. omari*, intermediate in *G. carapo* and shortest in *G coropinae*. This is compatible with the differences in the peak power frequency and the span of the power spectral density histogram (Figs. 1 and 6).

**Figure 7:**
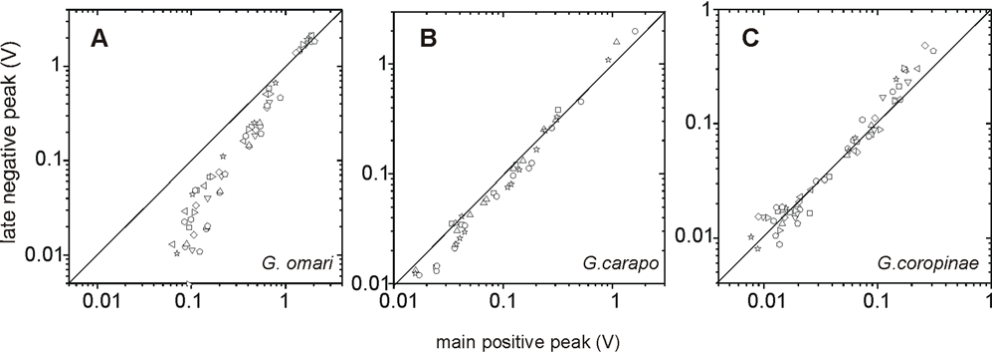
The correlations between the two main peaks indicate different responsiveness of the electrocytes. The late negative peaks (V_4_) recorded in air gap conditions from 6 regions of the body were plotted as a function of the main positive peaks (V_3_) recorded from the same 6 regions. It is shown that the relationship between these components is similar in different fish. Different symbols indicate different specimens (*G omari* N = 10; G. *carapo* N = 4; *G coropinae* N = 10). In *G. omari* V_3_ is generally larger than V_4_ while in *G carapo* and *G. coropinae* they are similar. Since the putative mechanism of V_4_ is the propagation of the action potential causing V_3_ to the opposite electrocyte face, changes in the slope indicate that the responsiveness of the electrocytes is different for each species-the lowest for *G. omari*, and the largest for *G. coropinae*.

Second, the electrocyte responsiveness appears to be larger in tropical species since: i) The late negative component (V_4ct_) was sharper in these two species than in *G. omari*; ii) The peak of V_4rct_ was well correlated toV_3rct_ having significantly different slopes for each species (*G. coropinae*, r^2^ = 0.96 , N = 24, p<0.0001*; G. carapo*, r^2^ = 0.98, N = 12, p<0.0001; *G. omari*, r^2^ = 0.81 N = 36, p<0.0001, Fig 7) and iii) V_5_ is absent in *G omari*.

The third and perhaps most striking difference between the two tropical species and G. omari was the presence of a positive slow wave (V_1ct_) generated at the central and tail regions of the fish body. This was observed in all specimens of *G. carapo* and in 3 out of 8 specimens of *G. coropinae* but was not observed in G. omari (Fig. 5A). It should be noted that the head to tail electric field generated by this component in *G. carapo*, overcomes the simultaneously occurring V_1r_ of opposite polarity _(_ fig 5B middle column). This yields as a net result the positive small P_−1_ described by Crampton [38]. This contrast with *G. coropinae* in which P_−1_ (sensu Crampton [38]) is the electric field generated by V_3r_ (fig 5B, right column).

## Discussion

Electric organ discharges are stereotyped effector acts which can be objects of holistic analyses that inform on their underlying control mechanisms. Combining different methods of analysis including far field, near field and air gap recordings we found that the fish body has multiple generators activated in a precise sequence for three species of *Gymnotus*. These studies, applied in the context of interspecific variation within *Gymnotus*, yielded information on the organization, function and evolution of electrogenesis.

Each component in the head to tail results from the sum of the activities at different regions of the fish body, weighted by a factor depending on the distance of the electrodes to the fish and on the constant of attenuation of each region. Earlier components, generated in small portions of the fish body by relatively small electromotive forces, have a relative small amplitude in the head to tail electric organ discharge, but are well defined in the near field. In contrast, the main complexes generated on the central and tail regions show high degree of synchronism between homologous components, and consequently, yield relative large amplitude components in the head to tail electric organ discharge. In addition, since the timing of each component at each site of the electric organ is not the same, components flowing in the same direction summate and those flowing in opposite direction subtract. Therefore, the order of the peaks in the head to tail electric organ discharge does not reflect neither similar spatial origin nor similar generation mechanism as can be clearly demonstrated comparing P_−1_ in *G. carapo* and *G coropinae*.

**Figure 8:**
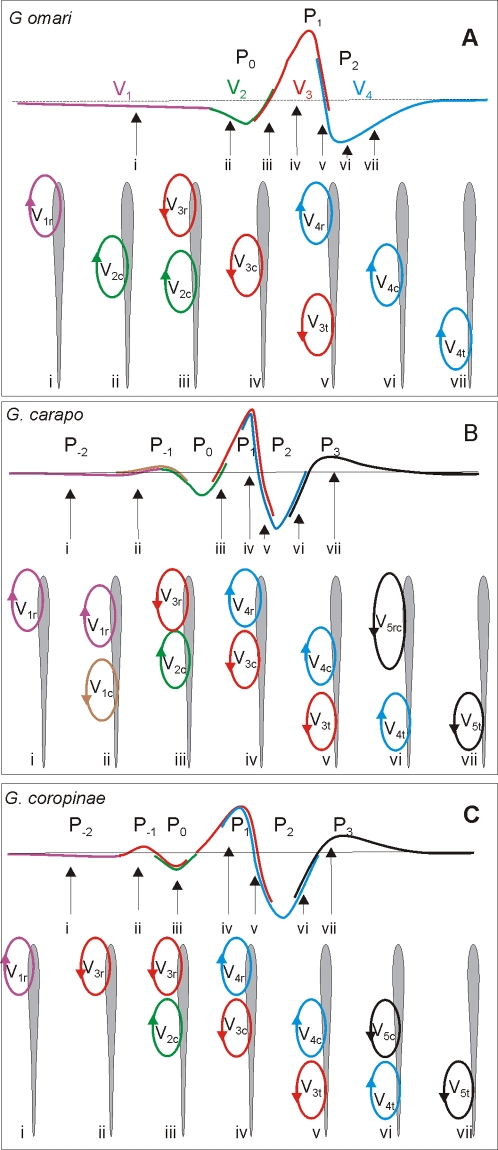
Present hypothesis on the formation of the head to tail electric organ discharge. In each box the head to tail waveform is represented in the top schematized trace where the participation of regional components in the head to tail is color coded. For the sake of generality we name each component combining a numeral sub index indicating the temporal order and a literal sub index indicating the spatial origin (r for rostral, c for central, and t for tail) . The schemata below represent using a color code the current generators at different regions of the fish body, at seven different times. *G. omari* is the simplest case: i) the earlier phase of P_0_ (V_1_ in the nomenclature of Trujillo-Cenóz et al [44]) corresponds to currents generated by V_1r_ (violet), ii) the late phase of P_0_ (V_2_ [44]) corresponds to the summated currents generated by V_2c_ (green) and the beginning of V_3r_ (red) since those have opposite direction their effects are contrary; the beginning of P_1_ (V_3_ [44]) is generated by the end of V_2c_ plusV_3r_, while the peak corresponds to V_3c_ plus V_3t_. The decay of P_1_ corresponds to V_3t_ plus the beginning of V_4r_ (red and blue traces superimposed) and P_2_ (V_4_) corresponds to V_4c_ plus V_4t_ (blue trace). In *G. carapo*: the correspondence is: i) P_−2_ to V_1r_ (violet) ; ii) P_−1_ to V_1r_ (violet) and V_1c_ (brown); iii) P_0_ to V_2c_ (green) and V_3r_ (red ) at the end of this wave; iv) the beginning and peak of P_1_ to V_3c_ (red ) and V_4r_ (blue); v) the decay of P_1_ to V_3t_ (red ), and V_4c_ (blue) ; vi) P_2_ to V_4t_ (blue) and V_5rc_ (black); and vii) P_3_ to V_5t_ (black). In *G. coropinae* the correspondence is: i) P_−2_ to V_1r_ (violet); ii) P_−1_ to V_3r_ (red); iii) P_0_ to V_2c_ (green) and V_3r_ (red ); iv) the growing phase and the peak of P_1_ to V_3c_ (red ) andV_4r_ (blue) ; v) the decay of P_1_ to V_4c_ (blue) and V_3t_ (red ); vi) P_2_ to V_5c_ (black)and V_4t_ (blue); vii) P_3_ to V_5t_ (black).

Taking into account the results obtained using the combined study of the near and far electric fields recorded in water and equivalent electromotive forces recorded when the fish is in the air we developed an hypothesis on how the different generators of each fish are represented in the far field for each species (Fig. 8).

### What do electric organ discharge patterns tell us about the organization of the electrogenic systems?

In all three species the rostral region of the fish's body is activated significantly earlier than the rest of the body. This regional discharge, which is similar in the three species, is characterized by a V_134r_ pattern (sometimes followed by a fifth component). The long lasting early negative component (V_1r_), and the positive component (V_3r_) together increase the relative weight of the lower frequency bands of the power spectra (upper histograms, Fig. 6). On the contrary, the central and caudal regions show V_234_ sequences that are similar in their polarity sequence to that seen in the rostral region, but occurring later, and in *G. carapo* and *G. coropinae* shorter in duration. The central and caudal region waveforms also show greater differences between species. These differences in the ranges and modes of the power spectral density histograms determine the species specificity of the electric organ discharge. It is important to note that the local electric organ discharges of the central regions are higher than those of the caudal regions. This is due to: a) the presence of a V_2c_ of similar duration to V_3c_ (see below), and; b) the lack of load in the air gap procedure which prevents a complete expression of V_4t_ (Rodriguez-Cattaneo, unpublished data).

In the case of a myogenic organ, Paccini's rule [53] states that the negativity indicates the active electrocyte face. With the known exception of *Malapterurus*, the innervated face is the first to be activated [54]. As in *G. omari*, early head negative waves (V_1r_ /V_2c_) must result from neural activation of rostral faces; while the main positive peaks at the tail (V_3t_) must result from the neural activation of caudal faces. In the three species, the presence of head negative waves without previous activity in the same region suggests the existence of rostrally innervated electrocytes and the presence of head positive waves without previous activity in other regions suggest the innervation of caudal faces. It is also likely that, as in *G. omari*, the main positive peak (V_3_ or P_1_) is likely to result from neural activation of caudal faces all along the electric organ. On the other hand, late negative peaks (V_4rct_) most likely result from action potential propagation from caudal to rostral faces as suggested by the amplitude correlation between regional V_3_ and V_4_.

One of the most important findings was a weak head positive V_1ct_ was always present in *G. carapo*, and sometimes present in *G. coropinae.* This component was never present in *G. omari* and may correspond to an (as yet), undescribed mechanism. Two explanations may account for the origin of this component. One is that the activity of the posterior electromotor nerve is strong enough to generate an externally observed signal (the presence of neural electro generation is already known in Apteronotidae [2] and this activity can be recorded locally in *G. omari* [7], [8]). Alternatively, and perhaps more interesting in the evolutionary sense, it is reasonable to hypothesize (by applying Paccini's rule) that there may be a set of electrocytes that are activated early, near the boundary between the central and caudal thirds of the body.

Finally, the different durations of the complex V_345ct_ in different species indicates differences in electrocyte excitability as well as in the timing of electrocyte excitation. Considering the hypothesis that components with sub index 4 are expressions of the same cellular mechanism (i.e. the propagation of the action potential from the caudal to the rostral faces of electrocytes [53], [45], [47]), their amplitudes are expected to be well correlated with the amplitude of the corresponding regional components with sub index 3 (as observed in Fig. 7). The steeper slopes of such linear relationship and the larger ratios between V_3_/V_4_ components in *G. coropinae* and *G. carapo* indicate that the rostral faces of their electrocytes are more responsive.

The late positive peak is a characteristic of *G. coropinae* and *G. carapo*. Curarization studies may be necessary to test their probable origin on the reverberant response of more responsive electrocytes to the caudal activation volley. The delay between V_2_ and V_3_ (determined by neural mechanisms) closely matches the delay between V_3_ and V_4_ (determined by electrocyte mechanisms). This indicates that the species specific differences in power spectra described here are not only due to changes in electrocyte responsiveness, but also to changes in neural coordination mechanisms.

### What do the electric organ discharge patterns tell us about the function of the electrogenic systems?

We have discussed in the first section the importance on near and far field analysis for understanding the electrolocating function of the electric organ discharge. Having this analysis in mind, one can discuss what kind of signals are represented in the near fields and far fields.

The near fields of the studied species show significant complexity, with different degrees of departure from a common pattern depending on the body region. Rostrally generated temporal patterns of electromotive force are more alike between species and have power spectra shifted to the low frequency range. They are characterized by a slow negative V_1r_ and a V_3r_ advanced in phase in reference to the V_3ct_, and a relatively poorly developed V_45r_. These waveforms are best represented in the local electric organ discharge at the rostral regions, close to where an electric fovea was described [52]. This observation, together with the similarity of food items found in the stomachs of several species of co-occurring *Gymnotus* from the Amazon basin (Crampton, pers. obs.) supports the hypothesis that the perceptual space for prey may be similar among closely related species. In contrast to their importance in the near field, rostrally generated components are poorly represented in the far field recordings. These recordings reflect very much the signals received by other sympatric fish sharing the same ecological habitats [38]. Thus the diversity expressed in the electromotive force patterns generated at the central and tail regions of the fish's body are translated to the far field recordings.

This supports the hypothesis that central and tail regions provide the species specificity of the communication carrier [39]. Ecologically co-occurring species of gymnotiforms (e.g. *G. carapo* and *G coropinae*) often exhibit non-overlapping ranges of far field power spectra suggesting that these species may be able to recognize and discriminate each other on the basis of frequency components of the electrocommunication carrier. However, in a higher diversity community from floodplain habitats of the Central Amazon, Crampton observed overlapping ranges of the power spectra among three of five co-occurring species [38]. Each of the five species was partitioned in a multivariate ‘signal space’ representing features of the head to tail electric organ discharge waveform, suggesting that these species may recognize and discriminate each other on the basis of both frequency and temporal components of the electrocommunication carrier.

### What are the phenotypic characters that the comparison between these species reveals as interesting from an evolutionary point of view?

Our results suggest at least three phenotypic characters of the electromotor system that are related to signal diversity in *Gymnotus*: a) the head negative sources at the rostral (V_1r_ ) and central (V_2c_) regions suggesting the presence of rostrally innervated electrocytes; b) the presence of an early smooth and positive wave at the tail of *G. carapo* (and variably in *G. coropinae*) suggesting a complex degree of spinal and peripheral synchronization of the discharge and c) the different relative importance of the non-neurally generated components V_45rct_, suggesting species specific variations of the electrocyte excitability.

The first character listed above, is the presence of an initial early negative component indicating the existence of rostrally innervated electrocytes at the head and abdominal regions of *G. coropinae, G. carapo,* and *G omari*. The presence of a pair of coordinated volleys of neural excitation on their opposite faces can be seen as an agonist antagonist pattern and may be an example of the selective benefits of increased complexity of central motor control. This contrasts with the monophasic discharge of some *Gymnotus* species (*G.cylindricus* and *G. maculosus*) from Central America. These fish lack early negative phases, have a less complex discharge, and probably possess a less complex neural network [42], [55].

The second character listed above, is the presence of a very distinct component in the central and caudal regions in *G. carapo* (V_1ct_). This component results in a species specific feature in the far field, and suggests the existence in this species of a) an additional early electrocyte activation volley that reach the tail region before the activation of V_3r_ and V_2c_ at more rostral regions, or b) the presence of a neural electrogenic mechanism in a genus traditionally consider as having a myogenic electric organ.

Finally, the different duration of the complex V_345_ indicates differences in electrocyte excitability, and the parallel adaptation of the corresponding electromotor control system. These differences are clearly significant at the tail region, reflecting the electrocyte responses characteristic of each species (the lowest responsiveness in *G. omari,* the highest responsiveness in *G. coropinae*).

### Conclusions

The main differences in the waveform between the three studied species suggest that electric organ discharge diversity in *Gymnotus* is based on: a) the appearance of neural coordination mechanisms for central triggering of multiple (but phase locked) volleys of activation of the electric organ; b) the double innervation of the electrocytes; and c) species specific responsiveness of electrocytes that are possibly based on their channel repertoire and shape. The rostral region of the three species generate similar profiles of electromotive force and local fields, while most of the species-specific differences are generated at the main body and tail regions of the fish. This supports a hypothesis of differential (although partially overlapped) origins of the electrolocation and electrocommunication carriers [39], [56]. Since the electric organ discharge's associated fields carry signals for electrolocation and communication, the species specificity and diversity of these fixed action patterns might be potentially related to food identification and recognition in the reproductive context [38]. Our results suggest that electrolocation or food identification is mediated by a carrier, or local electric organ discharge waveform, that is very similar across the three species, whereas electrocommunication or mate recognition may be mediated by a far field electric organ discharge that is quite diverse among the different species.

## Materials and Methods

All experiments were non invasive. Protocols were approved by the Instituto de Investigaciones Biológicas Clemente Estable and followed the guidelines of: a) the Comision Honoraria de Experimentación Animal (Universidad de la República) b) the Society for Neuroscience and c) the International Guiding Principles for Biomedical Research Involving Animals. The fish were maintained in individual aquaria and fed daily with insect larvae.

Animals were studied within 40 days of capture (mainly during the first two weeks) using several methods including far field recordings, near field recordings, and air gap measurements of the electromotive forces and internal resistances. Far field recordings were made in a temporary field laboratory from 15 reproductively mature *G. carapo* (total length range 182–340) and 15 mature *G. coropinae* in Suriname (total length range 100–138), and from 33 mature *G. omari* in Uruguay (total length range 121–220). Six *G. carapo* (length range 206–298 mm), 12 *G. coropinae* (length range 99–153 mm) and 15 *G. omari* (length range 123–253 mm) were used for detailed electric organ discharge analysis in the laboratory, including near field and electromotive force recordings.

### Fish and collecting sites

*Gymnotus carapo* is the type species of the genus. Until recently, many species of *Gymnotus* from throughout South America were indiscriminately assigned to *G. carapo*. For example, *G. omari*, in which the mechanisms for neural and peripheral organization were deeply studied [7], [8], [44]–[52], was for three decades incorrectly called *G. carapo*. However, Albert and Crampton's [57] redescription of *G. carapo* restricted this species to the Amazon and Orinoco basins, the coastal drainages of the Guyana, and some coastal basins of northeastern Brazil. *Gymnotus carapo* and *G. coropinae* were both originally described from Surinam, and here we present electric organ discharge data from populations of these species captured in northern Surinam. *Gymnotus coropinae* is a widely distributed species, occurring throughout most of the Amazon and Orinoco basins and the Guyanas [58]. It occurs sympatrically with *G. carapo* throughout much of its range, including in Surinam where the two species were collected in the same habitat at all sampled locations.

*Gymnotus carapo* and *Gymnotus coropinae* were captured from rainforest streams in northern Surinam, approximately 5° north of the equator. At this latitude the photoperiod varies from 11.8–12.4 hrs. Recorded mean daily air temperatures at the Paramaribo airport in 2006 exhibit a standard deviation of 1.5 from a mean annual value of 26.9°C (min 16.7, max 32.2 ) Water temperatures in rainforest streams, generally vary by less than approximately 3 °C over the diel cycle, and throughout the year in shaded, forest streams (J. Mol, pers.com.).

*G. coropinae* typically occurs only in small rainforest streams with low electrical conductivity (6–30 µScm^−1^), relatively high dissolved oxygen concentrations (>3 mg/l) and low pH (<6). These streams are usually well shaded by the rainforest canopy and contain cool water (ca. 24–27°C). *G. coropinae* hides during the day in submerged leaf-litter, marginal root masses, or in crevices in the substrate, and emerges to forage among underwater structures during the night.

*G. carapo* occurs syntopically with G. *coropinae*, but also occurs in a wider range of habitats, including shallow lakes and swamps, and systems with higher electrical conductivity (up to c. 200 µScm^−1^; J. Mol, pers. com.). In rainforest streams throughout most of Suriname, *G. carapo* and *G. coropinae* are found together but without other *Gymnotus*. A third species *G. anguillaris* was described from Northern Suriname, but is extremely rare, or locally extinct (J. Mol pers. com.), and was not captured during this study. However, several other gymnotiforms co-occur with *G. carapo* and *G. coropinae.* The following additional species were captured or observed during a two-week survey of rainforest stream habitats in the region by WGRC: *Electrophorus electricus*, *Hypopomus artedi, Brachyhypopomus beebei, Brachyhypopomus brevirostris, Brachyhypopomus* n. sp. 1 (‘*electropomus*’ sensu Sullivan [59]); *Brachyhypopomus* n. sp. 2, *Hypopygus lepturus, Sternopygus macrurus, Eigenmannia gr. virescens*. With the exception of *Hypopomus artedi*, which is restricted to the Guyana, these communities of gymnotiforms strongly resembled those in similar rainforest stream habitats from the Amazon basin. *Gymnotus omari* occurs in small streams and lagoons of the coastal drainages of Uruguay (but not in neighboring Brazil and Argentina where additional species of *Gymnotus* are known).

*G. omari* typically lives in dense aquatic vegetation such as water hyacinths. At approximately 35° S, *G. omari* occurs near the southernmost limit of the distribution of the order (Rio Salados, Argentina71). At this temperate latitude, the photoperiod varies from 9.8–14.5 hr and the seasons are well marked. Average high and low temperatures in mid-winter (July) are 14 and 6 °C respectively, and in mid-summer 28 and 17°C respectively (www.underground.com). Water temperatures in shallow water bodies frequented by *Gymnotus* typically vary from around 10–30°C during the year. *G. omari* does not co-occur with other species of gymnotiforms (including other *Gymnotus*) in coastal drainages, but co-occurs with *Brachyhypopomus pinnicaudatus*, *B. bombilla*, and *Eigenmannia* sp. in more northerly and westerly areas [60].

### Measurements and representation of electric organ discharge associated electric fields

#### Far field analysis

Far fields generated by the electric organ discharges were recorded as follows: Each fish was held in an aquarium filled with water from the capture locality and maintained at 27°C +/−0.2°C for at least 12 hours before the recording. All recordings were taken at 27°C +/−0.1°C, and each fish was allowed 5 minutes to acclimate to the aquarium. The recording arena was 80 long by 40 cm wide, and filled to 40 cm depth. Individual fish were placed within a nylon-mesh sock. This was supported by a mesh cradle suspended in mid-water (20 cm depth) and positioned equidistant from the tank ends and walls. Signals were captured using silver/silver-chloride electrodes placed at the tank ends, and with a ground contact in the center. The electrodes were connected to a wide-band AC-coupled differential amplifier (Signal Recovery 5113). Electric organ discharges were digitized using a National Instruments 6052E digitizer at a sampling rate of 250 kHz and a resolution of 16 bits. Electric organ discharges were not recorded from specimens with a history of damage to the caudal appendage. Measurements of peak power frequency and signal duration were made using custom-written MATLAB and Java software designed by W. Crampton. Electric organ discharge durations were calculated with the beginning and end of the electric organ discharge taken at a 1% threshold of the amplitude of the normalized dominant positive phase. Spectral power density plots were calculated from 65,536-point Fast Fourier Transform.

#### Near field analysis

Electric fields produced by the electric organ discharge were recorded with the fish resting in the middle of a net pen running between the center of the narrow faces (28 cm) of a plastic tank (45×26 cm filled with water up to 4 cm depth, with conductivity 30 µScm^−1^, temperature 24°C). The back and forth movements of fish were minimized using stitches to adjust the net to body length. We used two different procedures: (i) the longitudinal electric organ discharge fields were recorded using two silver/silver-chloride electrodes, each placed at the center of each narrow face of the tank one facing the head and the other facing the tail; (ii) the near field recordings were measured using a specially designed probe placed close to the skin of the fish at different points along its body. The latter technique (described in detail elsewhere [39], [52]) was used to record local potential gradients equivalent to orthogonal components of the local electric field vector at that point. The probe was constructed from three wires insulated except at their tips Active electrodes were oriented along horizontal orthogonal axes (longitudinal *x*, lateral *y* ) intersecting at the point where the reference was placed. Their tips were 2.5 mm from the intersection of the axes, and the orientation was parallel to the longitudinal and horizontal axes of the fish, with the reference electrode facing the point on the skin under investigation. We recorded local electric fields at equally spaced points (5 mm steps) along a parasagittal line passing 2 mm from the nearest point on the fish skin surface at middle fish height. The voltage difference between each of the active electrodes and the reference electrode was measured using high-input impedance, high-gain differential amplifier (10 Hz to 20 kHz band-pass filter). Recorded waveforms were sampled (50 kHz or more depending the number of channels recorded, 16 bits) and displayed on a computer screen. Voltage measurements were considered to be proportional to the voltage gradient along the orthogonal axes and, therefore, to the horizontal components of the local electric organ discharge. Each local field orthogonal component was expressed in V.cm^−1^.

### Measurements of the source parameters

To evaluate the spatio-temporal pattern of equivalent electromotive force for the fish body we used the air-gap technique [7], [9] which consist of the simultaneous recording of the voltage drop generated by different portions of the fish's body when isolated in air. Fish were suspended in air using a custom made apparatus that holds the fish as in a grill. The wires in contact with the skin were perpendicular to the main axis of the body, one at each extreme of the fish and the other at the limits of the explored regions. For the purpose of this report three regions of the fish body were considered: a) the head and abdominal region (the rostral quarter of the fish in *G. carapo*, and the rostral two sevenths in the other species), the tail region (the caudal quarter of the fish in *G. carapo*, and the caudal two sevenths in the other species), and the central region (the rest of the fish's body).

Voltages recorded between pairs of wires were amplified to reach adequate amplitude for similar quantization (always larger than 8 bits) and sampled at 25 kHz. In the air gap condition, there was no load, so voltage recordings are good estimators of the equivalent electromotive forces generated by different portions of the fish's body when the electric organ is activated. Data were presented as time and frequency domain functions. For time domain analysis, data were aligned taking as a reference the head to tail electromotive force recording, equivalent to the sum of all recordings from the different portions of the fish. The amplitude and timing of each identified component was then directly measured. Fast Fourier transforms (in house routine) were calculated from 10 ms traces and spectral power density histograms were calculated with a bin of 100 Hz.
